# Modification of Mechanical Properties in Directed Energy Deposition by a Static Magnetic Field: Experimental and Theoretical Analysis

**DOI:** 10.3390/ma14185190

**Published:** 2021-09-09

**Authors:** Aleksandr M. Filimonov, Oleg A. Rogozin, Oleg N. Dubinin, Yulia O. Kuzminova, Anastasia A. Shibalova, Ilya V. Okulov, Iskander S. Akhatov, Stanislav A. Evlashin

**Affiliations:** 1 Center for Design, Manufacturing & Materials, Skolkovo Institute of Science and Technology, 121205 Moscow, Russia; Al.Filimonov@skoltech.ru (A.M.F.); O.Rogozin@skoltech.ru (O.A.R.); O.Dubinin@skoltech.ru (O.N.D.); Yulia.Kuzminova@skoltech.ru (Y.O.K.); I.Akhatov@skoltech.ru (I.S.A.); 2 World-Class Research Center ”Advanced Digital Technologies,” State Marine Technical University, 190121 Saint-Peterburg, Russia; 3 Institute of Nanotechnology of Microelectronics of Russian Academy of Science, 119991 Moscow, Russia; nastia0694@gmail.com; 4 Leibniz Institute for Materials Engineering—IWT, 28359 Bremen, Germany; i.okulov@iwt.uni-bremen.de

**Keywords:** directed energy deposition, Marangoni effect, magnetohydrodynamics, Seebeck effect, thermoelectric magnetic convection

## Abstract

The superimposed magnetic field affects the microstructure and mechanical properties of additively manufactured metal parts. In this work, the samples were fabricated from Inconel 718 superalloy by directed energy deposition under a 0.2 T static field. The magnetohydrodynamic 1D model is proposed for the estimation of a fluid flow inside a molten pool. According to the theoretical predictions, the fluid flow is slightly decreased by an applied field. The estimated thermoelectric magnetic convection in the mushy zone is shown to be negligible to change in subgrain size, but enough to reduce the hard-to-dissolve Nb-rich phase, thereby improving the average ultimate elongation from 23% to 27%. The obtained results confirm that an external static magnetic field can modify and enhance the mechanical properties of additively manufactured materials.

## 1. Introduction

The physical properties of additively manufactured (AM) material via directed energy deposition (DED) attract interest in various industries [1,2,3]. Nevertheless, both engineers and researchers struggle against uncontrollable multiscale physical processes, whose consequences can be mitigated by utilizing the magnetic field (MF) [4,5,6], ultrasound [7], preheating [8], etc.

The hydrodynamics at the molten pool scale are usually controlled by the dominant Marangoni force and recoil pressure [9,10,11,12]. The Marangoni convection is caused by a gradient of surface tension that is a function of concentration and temperature. The motion of the electrically conductive medium in the MF induces an electric current inside it. This phenomenon is called the magnetohydrodynamic (MHD) effect. The emerging Lorentz force dampens fluid convection [13,14], thereby reducing the residual porosity.

In a mushy zone, the temperature gradient near the liquid–solid interface generates the thermoelectric (TE) current (Seebeck effect) [15,16,17]. Depending on the applied MF and the TE current density, a thermoelectric magnetohydrodynamic (TEMHD) force arises [6]. The resulting interdendritic TEMHD convection can provoke the columnar to equiaxed transition (CET) and alter the solute distribution, thereby changing the subgrain (cell) microstructure, which in turn affects the overall mechanical properties [18,19]. At low solidification rates, a fluid flow in the interdendritic region is controlled by the buoyancy-driven force based on the thermal and solutal gradients, leading to a density difference [19,20]. However, in AM technology, the buoyancy-driven force becomes negligible.

Du et al. [14] found that applying a 0.12 T vertical static MF in laser powder bed fusion (LPBF) increases the ultimate strength by 23% and elongation by 32% in processed AlSi10Mg alloy. They also showed that the number of columnar grains decreases by 44% when exposed to an MF, while the dendrite arm spacing is reduced only slightly. In contrast, Du et al. [5] established that the cell spacing is increased at a 0.1 T MF during the DED for Inconel 718 superalloy. Wang et al. [21] showed that an applied 1.8 T MF leads to enhanced grain alignment along the preferable direction without changing the average size for Inconel 718 superalloy. Moreover, they showed that the TEMHD convection drastically increases the dissolution of hardly soluble elements by removing the brittle Laves phases. Liu et al. [22] demonstrated a 22% increase in ultimate elongation of fabricated Inconel 718 superalloy using 0.08 T electromagnetic stirring with 50 Hz frequency. Additionally, they observed that the Vickers microhardness is increased by 15%, and the volume fraction of the Laves phases is decreased by 56%.

Although the MF effects on the structural and mechanical properties of AM material are widely studied, and some results have already shown significant benefits in material performance, the obtained experimental outcomes for the cell spacing are ambiguous. Moreover, there is currently no thorough theoretical analysis of the MF influence on the evolution of the AM material microstructure.

This study has a twofold purpose. Firstly, to analyze a 0.2 T vertical and a 0.15 T horizontal MF influence on the material performance of fabricated Inconel 718 superalloy by the DED. Secondly, to explain the observed results using theoretical analysis.

## 2. Theoretical Methods

### 2.1. Fluid Flow in the Molten Pool

The following equations, written in conventional SI units, describe the flow of incompressible viscous electrically conducting fluid in the presence of an MF [23,24]:

(1) $$\begin{array}{c} \nabla \cdot v=0,\;\nabla \cdot B=0 \end{array}$$

(2) $$\begin{array}{c} \frac{dB}{dt}=\left(B\cdot \nabla \right)v+\frac{1}{\mu_{0}\sigma }\nabla^{2}B \end{array}$$

(3) $$\begin{array}{c} \rho \frac{dv}{dt}=\eta \nabla^{2}v-\nabla p-\frac{1}{\mu_{0}}B\times \nabla \times B \end{array}$$

where v is the velocity of the fluid flow, B is the magnetic field, $\mu_{0}$ = 1.256 × 10^−6^ H m^−1^ is the vacuum permeability, σ is the electrical conductivity, ρ is the density, η is the dynamic viscosity, *p* is the internal pressure.

The above system of equations supposes that the electric current density j is derived via generalized Ohm’s law [16]:

(4) j=σ(E+v×B−S∇T)

where E is the electric field intensity, *S* is the absolute TE power of the medium or the absolute Seebeck coefficient, *T* is the temperature.

Finally, it is assumed that the magnetic permeability μ= 1 and the displacement current is much less than the electric current due to the low-frequency (non-relativistic) assumption, thereby eliminating the polarization of the medium. The following boundary conditions are valid at the gas–liquid interface [24]:

(5) $$\left(p_{G}-p_{L}+\gamma \nabla \cdot \hat{n}\right)\cdot \hat{n}=\left(\Theta_{G}-\Theta_{L}\right)\cdot \hat{n}+\hat{n}\times \nabla \gamma \cdot \hat{n}$$

where subscripts G and L correspond to the gas and liquid quantities, respectively, γ is the surface tension, $\hat{n}$ is the unit normal directed into the gas, and

(6) $$\Theta =\eta (\nabla v+{\left(\nabla v\right)}^{T})$$

is the viscous stress tensor. In the case of a temperature-dependent surface tension, γ=γ(T), $\nabla \gamma =\gamma^{\prime }\nabla T$. Additionally, conditions

(7) $$v_{L}=v_{G},\;B_{L}=B_{G}$$

are imposed in the case η>0 and σ<∞.

The general boundary conditions at the liquid–solid interface are derived by integrating Equation (4) around the narrow loop straddling the interface [15]:

(8) $$v_{L}=v_{S},\;B_{L}=B_{S},\;\hat{n}\left(\frac{j_{S}}{\sigma_{S}}-\frac{j_{L}}{\sigma_{L}}-\left(S_{L}-S_{S}\right)\nabla T\right)\times \hat{n}=0$$

where subscript S corresponds to the solid quantity, $j_{S/L}$ are obtained from Maxwell’s equation [15,25]:

(9) $$j_{S/L}=\frac{{\left(\nabla \times B\right)}_{y}}{\mu_{0}}=\frac{1}{\mu_{0}}\frac{\partial B_{x}}{\partial z}$$

**Figure 1:**
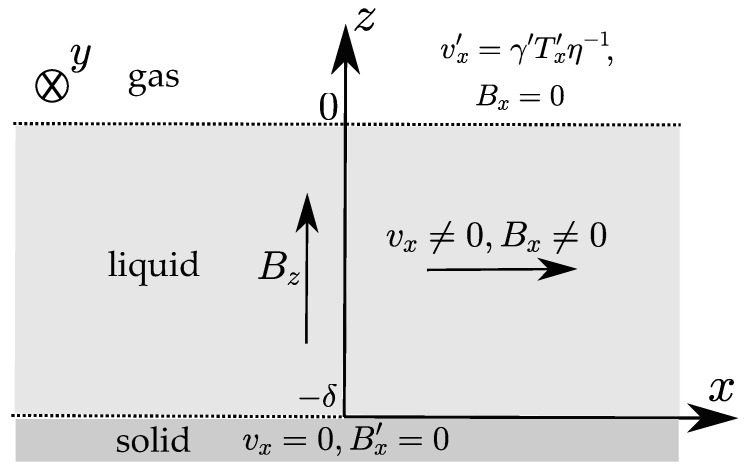
The problem geometry under the vertical static MF.

Let us consider a simplified 1D model of the molten pool under the static vertical MF $B_{z}=B_{1}$ as a unidirectional flow along the *x* axis between two infinite parallel surfaces: the upper one (z=0) corresponds to the gas–liquid interface, and the lower one (z=−δ) corresponds to the liquid–solid interface, where the fluid is resting (Figure 1). The uniform temperature gradient $T_{x}^{\prime }$ exists along the gas–liquid interface. The ambient pressure is equal to $p_{0}$.

Under assumptions d/dt=0, d/dx=0, d/dy=0, $v_{y}=0$, $v_{z}=0$, and $B_{y}=0$, the set of Equations (1)–(3) reduces to

(10) $$\frac{dB_{z}}{dz}=0,\;\frac{dv_{x}}{dz}=-\frac{1}{\sigma \mu_{0}B_{z}}\frac{d^{2}B_{x}}{dz^{2}},\;\frac{d^{2}v_{x}}{dz^{2}}=-\frac{B_{z}}{\eta \mu_{0}}\frac{dBx}{dz},\;\frac{d(2\mu_{0}p+B_{x}^{2})}{dz}=0$$

The general solution of Equation (10) is given as

(11) $$\begin{array}{c} v_{x}=\frac{1}{\tau }\left(C_{1}\cosh\left(\tau z\right)+C_{2}\sinh\left(\tau z\right)+C_{3}\right) \end{array}$$

(12) $$\begin{array}{c} B_{x}=-\frac{\mu_{0}\eta }{B_{z}}\left(C_{1}\sinh\left(\tau z\right)+C_{2}\cosh\left(\tau z\right)+C_{4}\right) \end{array}$$

(13) $$\begin{array}{c} B_{z}=C_{5},\;p=\frac{B_{x}^{2}}{2\mu_{0}}+C_{6} \end{array}$$

where $\tau =B_{z}\sqrt{\sigma /\eta }$ and $C_{i}$ are some specific constants. The boundary conditions (5) and (7) take the form

(14) $$\eta \frac{dv_{x}}{dz}=\gamma^{\prime }T_{x}^{\prime },\;p=p_{0},\;B_{z}=B_{1},\;B_{x}=0$$

at z=0. Here, it is assumed that the gas viscous stress tensor is negligible. In addition, due to the absence of the electric current in the gas, $\nabla \times B_{G}=0$, which defines the field to a constant. However, for the sake of certainty, we zero it out.

The remaining boundary conditions (8) and (9) at z=−δ are simplified to

(15) $$v_{x}=0,\;\frac{dB_{x}}{dz}=0$$

Here, it is supposed that the longitudinal temperature gradient is assumed to also be negligible. The constants are given by

(16) $$C_{1}=C_{2}\tanh\left(\tau \delta \right),\;C_{2}=\frac{\gamma^{\prime }T_{x}^{\prime }}{\eta },\;C_{3}=0,\;C_{4}=-C_{2},\;C_{5}=B_{1},\;C_{6}=p_{0}$$

Therefore, the general solution (11)–(12) takes the form

(17) $$\begin{array}{c} v_{x}=\frac{\gamma^{\prime }T_{x}^{\prime }}{\eta \tau }\cosh\left(\tau z\right)(\tanh(\tau \delta )+\tanh(\tau z)) \end{array}$$

(18) $$\begin{array}{c} B_{x}=\frac{\mu_{0}\gamma^{\prime }T_{x}^{\prime }}{B_{1}}\sinh\left(-\tau z\right)\left(\tanh\left(\tau \delta \right)+\tanh\left(\frac{\tau z}{2}\right)\right) \end{array}$$

which can be nondimensionalized as

(19) $$\begin{array}{c} {\hat{v}}_{x}=\frac{\cosh(\mathrm{Ha}\hat{z})}{\mathrm{Ha}}\left(\tanh\left(\mathrm{Ha}\right)+\tanh\left(\mathrm{Ha}\hat{z}\right)\right) \end{array}$$

(20) $$\begin{array}{c} {\hat{B}}_{x}=\frac{\sinh(-\mathrm{Ha}\hat{z})}{\mathrm{Ha}}\left(\tanh\left(\mathrm{Ha}\right)+\tanh\left(\frac{\mathrm{Ha}\hat{z}}{2}\right)\right) \end{array}$$

according to the following relations:

(21) $$v_{x}=v_{0}{\hat{v}}_{x},\;B_{x}=B_{0}{\hat{B}}_{x},\;z=\delta \hat{z},\;\mathrm{Ha}=\tau \delta$$

where Ha is the Hartmann number, which determines the ratio between the magnetic and viscous forces, $v_{0}$ and $B_{0}$ are the characteristic velocity and induced MF, respectively, given by

(22) $$v_{0}=\frac{\gamma^{\prime }T_{x}^{\prime }\delta }{\eta },\;B_{0}=\mu_{0}v_{0}\sqrt{\sigma \eta }$$

**Figure 2:**
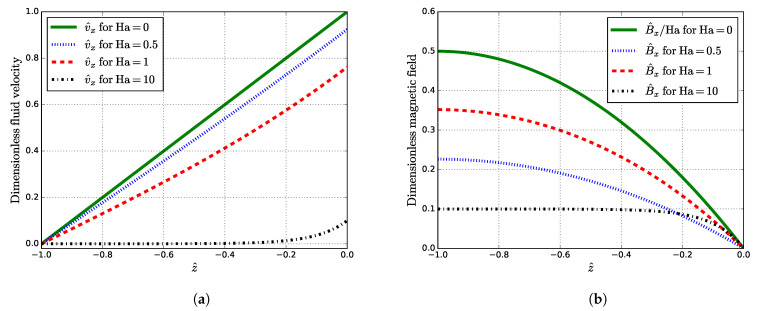
Dimensionless quantities: (**a**) velocity ${\hat{v}}_{x}$ and (**b**) induced MF ${\hat{B}}_{x}$, given by (19) and (20), as functions of $\hat{z}$ For small Ha, ${\hat{v}}_{x}=1+\hat{z}+O$(Ha^2^) and ${\hat{B}}_{x}=-\mathrm{Ha}\hat{z}(\hat{z}/2+1)+O$(Ha^3^).

The obtained solutions are shown in Figure 2.

As shown in Figure 2a, the value of ${\hat{v}}_{x}$ is dampened as Ha grows. As the Ha number increases, ${\hat{B}}_{x}$ decays (Figure 2b). Notice that at Ha=1, the induced MF at the liquid–solid interface is slightly higher than at Ha=0.5. Velocity ${\hat{v}}_{x}\left(\hat{z}\right)$ and induced MF ${\hat{B}}_{x}\left(\hat{z}\right)$ reach their maxima at $\hat{z}=0$ and $\hat{z}=-1$, respectively, where their values are

(23) $${\hat{v}}_{x,\max}={\hat{v}}_{x}\left(\hat{z}=0\right)=\frac{\tanh(\mathrm{Ha})}{\mathrm{Ha}},\;{\hat{B}}_{x,\max}={\hat{B}}_{x}\left(\hat{z}=-1\right)=\frac{1-\mathrm{sech}(\mathrm{Ha})}{\mathrm{Ha}}$$

Furthermore, the change in the fluid velocity at the liquid–solid interface ($\hat{z}=-1$) is proportional to the viscous force that acts tangentially on the solid surface:

(24) $${\left.\frac{d{\hat{v}}_{x}}{d\hat{z}}\right|}_{\hat{z}=-1}=\mathrm{sech}(\mathrm{Ha})$$

**Figure 3:**
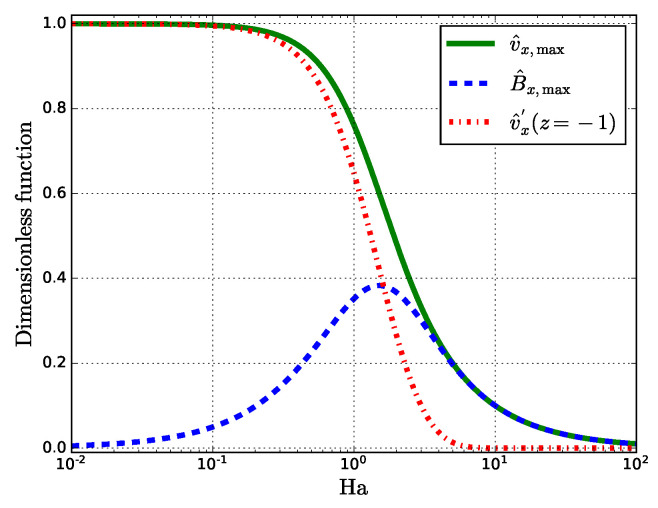
Semi-log plot of ${\hat{v}}_{x,\max}$, ${\hat{B}}_{x,\max}$, and ${\hat{v}}_{x}^{\prime }$ versus Ha.

Relations (23) and (24) are shown in Figure 3.

Since ${\hat{v}}_{x,\max}$ is a monotonically decreasing function of Ha, it should be inferred that the induced Lorentz force, regardless of its strength, has a damping effect and brakes the fluid flow. The behavior of the induced MF near Ha=1 (Figure 2b) can be explained by reaching a maximum with a subsequent drop. For small Ha, the maximum induced MF ${\hat{B}}_{x,\max}$ is Ha/2 that gives the following relation to the external static MF $B_{1}$:

(25) $$\frac{B_{x,\max}}{B_{1}}=\frac{B_{0}\mathrm{Ha}}{2B_{1}}=\frac{\mu_{0}v_{0}\sigma \delta }{2}$$

### 2.2. Fluid Flow in the Mushy Zone

**Figure 4:**
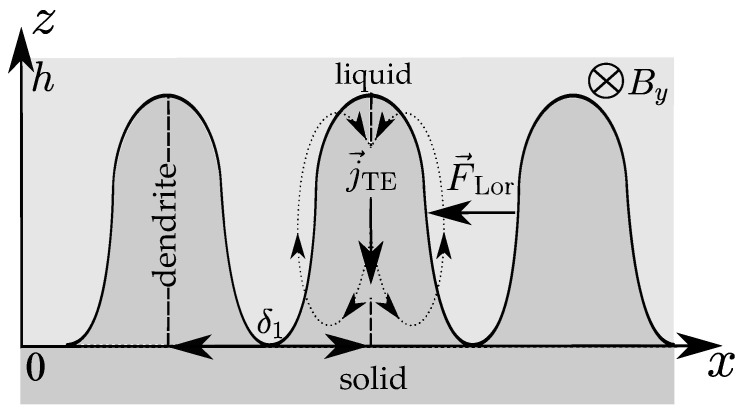
The problem geometry under the horizontal static MF.

The 2D picture of the fluid flow near the liquid–solid interface (a mushy zone) under the static horizontal MF $B_{y}=B_{2}$ and a longitudinal temperature gradient $T_{z}^{\prime }$ is schematically shown in Figure 4. The primary dendrite arm spacing (PDAS) $\delta_{1}$ and the thickness of the mushy layer *h* limit the interdendritic fluid flow.

According to the approach proposed by Lehmann et al. [16], Darcy’s equation can accurately describe the behavior of the fluid flow:

(26) $$\eta \frac{U}{K}=F_{B}+F_{L}$$

where U is fluid velocity, *K* is the permeability of a mushy zone, $F_{B}$ and $F_{L}$ are the buoyancy force and the Lorentz force per unit volume, respectively, which are given by [16,17,18]:

(27) $$\begin{array}{c} F_{B}=\rho_{L}\beta_{C}g\Delta C \end{array}$$

(28) $$\begin{array}{c} F_{L}=j_{L}\times B \end{array}$$

where $\beta_{C}$ is the solutal expansion coefficient, ΔC is the difference of the eutectic concentration $C_{e}$ and initial concentration of the alloying element $C_{o}$.

The electric current density in the liquid $j_{L}$ can be found from the boundary conditions (8). Additionally, the condition for conserving the total current is added [15,26]:

(29) $$j_{L}=-PT_{z}^{\prime }\sigma_{\mathrm{eff}}\varepsilon_{S}$$

where

(30) $$P=S_{L}-S_{S},\;\sigma_{\mathrm{eff}}=\frac{\sigma_{S}\sigma_{L}}{\sigma_{L}\varepsilon_{L}+\sigma_{S}\varepsilon_{S}}$$

where $\varepsilon_{S/L}$ is the volume fraction of the particular phase. It should be emphasized that Equation (27) is obtained according to the Oberbeck–Boussinesq approximation, where the thermal expansion coefficient $\beta_{T}$ ($∼10^{-4}$$/K^{-1}$ [27]) is omitted due to its smallness compared to $\beta_{C}$ ($∼10^{-2}$/wt.% [17]).

Owing to the specific liquid–solid distribution in the mushy zone, the definition of the permeability value is complicated. Moreover, since the structure of AM material mainly consists of columnar dendrites, the permeability should be considered anisotropic instead of the generally accepted Blake–Kozeny equation for the equiaxed dendrites [28]. Chamsri et al. [29] adapted the empirical Kozeny–Carman equation for the vertical columnar dendrites (cylindrical case):

(31) $$K=\frac{2.0128\varepsilon_{L}^{3}\delta_{1}^{2}}{4{\left(1-\varepsilon_{L}\right)}^{2}}$$

According to the lever rule [30,31], the liquid fraction $\varepsilon_{L}$ can be obtained from phase diagrams, assuming the temperature grows linearly along dendrites [32,33]. Moreover, the liquid fraction can be accurately predicted using kinetic models, but this is beyond the scope of this study. Thus, for simplicity, we take $\varepsilon_{S}=\varepsilon_{L}=0.5$.

Assuming that $F_{B}$ is negligible compared to $F_{L}$ (the corresponding Lorentz–buoyancy ratio κ will be presented below in Section 4.1), Equation (26) shows that the direction of the total force is determined generally by $F_{L}$. Thus, the magnitude of the velocity *U* is as follows:

(32) $$U=\frac{KB_{2}PT_{z}^{\prime }\sigma_{\mathrm{eff}}}{2\eta }$$

Additionally, the Péclet number (Pe), which determines the ratio between the convection velocity and the rate of diffusion, is as follows [16]:

(33) $$\mathrm{Pe}=\frac{\delta_{1}U}{D}$$

where *D* is the diffusion coefficient.

## 3. Experimental Methods

### 3.1. Sample Preparation

**Figure 5:**
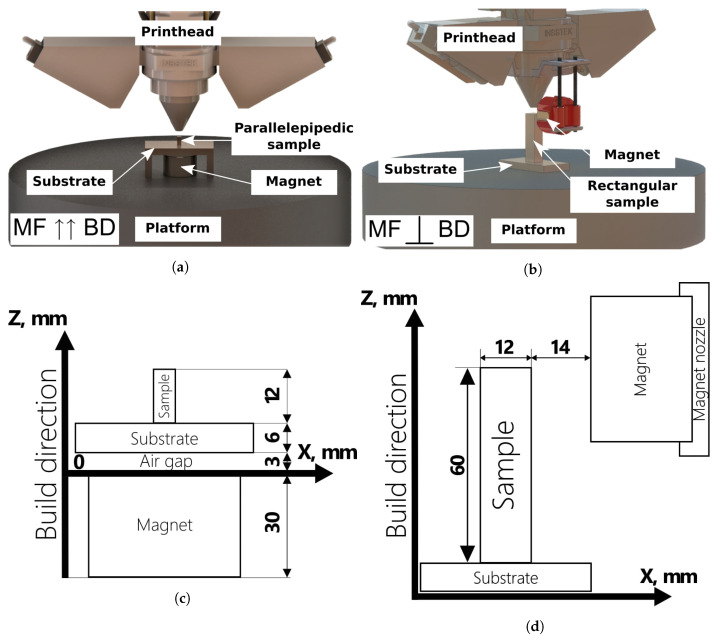
Experimental setups for 3D printing and their schematic illustrations for the case of vertical (**a**,**c**) and horizontal (**b**,**d**) MF directions.

Inconel 718 (Carpenter Powder Products Inc., Bridgeville, PA, USA) powder was used as a feedstock material, which has a particle size distribution of d_10_ = 45 μm and d_90_ = 150 μm. The DED technology, which is implemented through the direct metal tooling (DMT) InssTeK MX-1000 printer with an ytterbium fiber laser with a power of 2 kW and a wavelength of 1070 nm (InssTeK DMT Metal AM Technology Specialist, Daejeon, Korea), was used for the two types of sample manufacturing in a protective argon atmosphere. The parallelepipedic and rectangular parts with a 6 × 6 × 12 mm^3^ size and a 20 × 60 × 12 mm^3^ size were printed in vertical MF (the applied MF $B_{1}$ coincides with the build direction) at corresponding volume energy densities (Figure 5a,c). In addition, the same rectangular parts were fabricated in the horizontal MF using the developed magnetic nozzle (the applied MF $B_{2}$ is normal to the build direction) (Figure 5b,d). The corresponding optimal laser-related settings, such as the laser power and laser spot diameter, and scan-related parameters, such as the scan speed, hatch spacing, and other printing parameters, are shown in Table 1. The scan strategy consisted of square and rectangular patterns rotated at intervals of 90° in each *x*-*y* plane. An additional laser pause of 5 s between rotations was provided to ensure a smoother surface and remove corner swellings due to overheating. Additionally, to avoid structural changes in the magnet that can cause field instability and amplitude attenuation, an 3 mm air gap was provided between the upper part of the magnet surface and the lower part of the substrate. Additionally, the magnet surface was shielded by copper foil, reflecting approximately 95% of the incident infrared radiation at a wavelength of 1070 nm.

The as-deposited parallelepipedic samples were sliced along and across (half-height) the build direction using the Accutom-100 cutting machine (Struers, Ballerup, Denmark). The sample mounting was performed via a TechPress 2 machine (Allied Corp., Rancho Dominguez, CA, USA) and then ground using 800-grit SiC via a MetPrep 3 machine (Allied Corp.). The final polishing was completed with a 40 nm colloidal silica (OP-U) suspension on a porous neoprene cloth with the applied force of 30 per sample and a dwell time of 30 (Allied Corp.). All polished samples were cleaned by water and air-blasting.

The flat dog-bone specimens were cut from rectangular samples for the tensile testing using the Mitsubishi MV-1200 (MV1200R) Advance wire electrical discharge machine (MC Machinery Systems, Inc., Elk Grove Village, IL, USA). The geometry of the samples was established by the American Society for Testing and Materials (ASTM) E8 standard. The working surfaces of cut-out samples were ground using 1000-grit SiC (Allied Corp.).

### 3.2. External Magnetic Field

**Figure 6:**
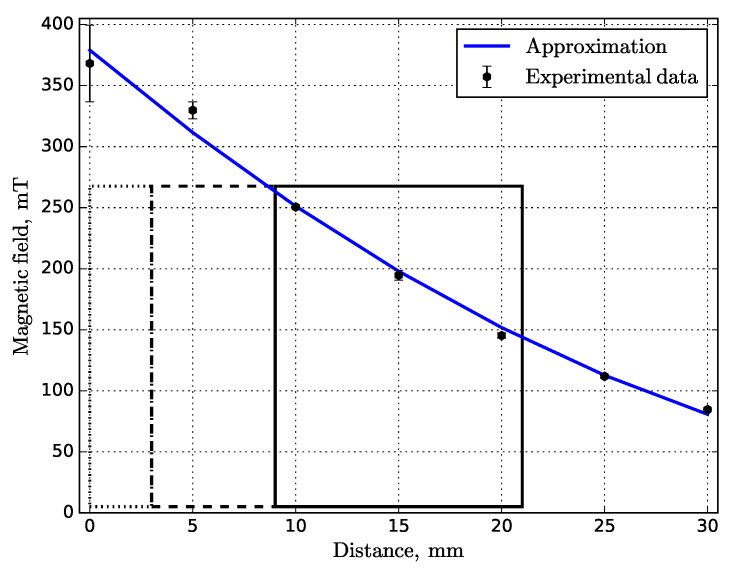
MF versus the distance from the top of the magnet surface. The corresponding rectangular regions are as follows: air gap (dotted), substrate (dashed), printed sample (solid). The average value of the vertical MF $B_{1}=203\;mT$.

The static MF was generated by the cylinder permanent neodymium Nd_2_Fe_14_B N52 magnet with a diameter of 50 mm and a height of 30 mm. The MF induction was measured using the Aktakom ATE–8702 magnetic meter (gaussmeter) with a maximum measurement of 3 T in the DC range (Lutron Electronic Enterprise Co., Ltd., Taipei, Taiwan). Additionally, to verify that the decrease in MF induction is negligible until a temperature threshold (maximum operating temperature) is reached, the measurements were conducted at room temperature and close to a maximum declared temperature (0.75$T_{\max}$ = 60 °C. The result obtained for the MF profile is shown in Figure 6.

### 3.3. Microstructure and Mechanical Properties Characterization

A Thermo Scientific Helios G4 PFIB UXe microscope (Thermo Fisher, Waltham, MA, USA) equipped with the Octane Elite Super detector (EDAX Inc., Mahwah, NJ, USA) was used for energy-dispersive X-ray spectroscopy (EDX), only for as-deposited parallelepipedic samples (front view). The mapping of the investigated sample surface was performed near the center of samples to obtain a more accurate result of the elemental distributions. The nitrogen and oxygen content was determined using a LECO TC-136 determinator (LECO Corporation, St. Joseph, MI, USA) for the fabricated sample under no and horizontal superimposed MF conditions. The hydrogen content was measured using an ELTRA ONH-2000 analyzer (Eltra GmbH, Haan, Nordrhein-Westfalen, Germany). According to the scanning electron microscopy (SEM) images taken with a Quattro S (Thermo Fisher), the average cell (subgrain) size was estimated by the mean linear intercept method using the open-source image processing software ImageJ 1.53e (Bethesda, MD, USA) for both views.

The front view of the fabricated sample under no and superimposed MF conditions was analyzed with X-ray diffraction (XRD) using a Bruker D8 ADVANCE (Bruker Corporation, Billerica, MA, USA) diffractometer with CuKα radiation (with a wavelength of 1.5418 Å) over a 2θ range between 30° and 100° at room temperature. The step size and dwell time were 0.03° and 100 s, respectively.

The parallelepipedic samples were investigated at room temperature for the planar porosity and microhardness in *x*-*y* and *x*-*z* planes. The planar porosity was determined using Axio Scope.A1 optical microscopy (Carl Zeiss AG, Jena, Germany) with Thixomet Pro software V 3.0.0048 (Thixomet Company, Saint Petersburg, Russia) and analyzed based on the ASTM E1245-03.

The microhardness was measured by microindentation testing using the Metrotest Vickers microhardness Tester ITV-1-AM (Metrotest LLC, Neftekamsk, Russia). The Vickers pyramid diamond indenter was used with an expansion angle of 136°. The microhardness measurements in the *x*-*z* plane included 24 points on each sample in the equivalent four regions with a width of ∼2 ÷ 3 mm. The approximate vertical distance between the measurement points was at least 10 ÷ 15 characteristic stamp sizes (∼750 μm), the applied load was 3N, and the creep time was 10 s. The microhardness measurements in the *x*-*y* plane included 10 points per sample with the same parameters of indentation described above.

The cut-out flat dog-bone specimens were subjected to tensile testing (gauge length was 32.0 ± 0.1 mm) at room temperature using the Instron 5969 Tensile Strength Tester (Illinois Tool Works Inc., Glenview, IL, USA) with a 10^−3^ s^−1^ strain rate in the build direction (vertical samples) and across (horizontal samples).

## 4. Results and Discussion

### 4.1. Theoretical Analysis

Let us use Equations (17), (18) and (32) for the numerical characterization of the fluid flow and induced MF for Inconel 718 superalloy. The physical properties of the used material and other parameters are presented in Table 2.

The temperature gradients $T_{x}^{\prime }$ and $T_{z}^{\prime }$ are defined as follows:

(34) $$T_{x}^{\prime }=\frac{T_{b}-T_{m}}{\delta },\;T_{z}^{\prime }=\frac{T_{\mathrm{tip}}-T_{\mathrm{root}}}{h}\approx \frac{T_{m}-T_{e}}{\delta_{1}}$$

where $T_{b}$ and $T_{m}$ are the boiling and melting temperatures, respectively, $T_{\mathrm{tip}}$ is the dendrite tip temperature, $T_{\mathrm{root}}$ is the melting point of the last interdendritic liquid or the eutectic temperature $T_{e}$ [18].

**Figure 7:**
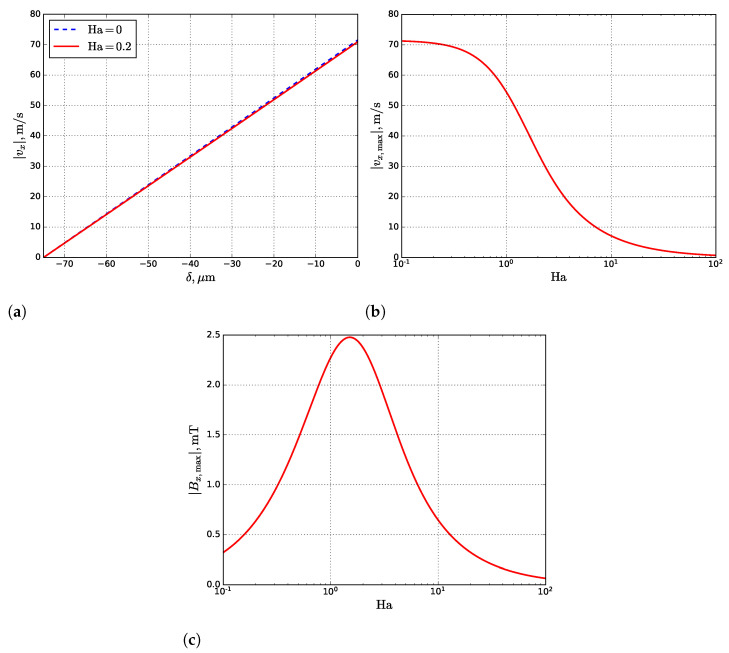
The profiles of fluid velocity (**a**) inside the molten pool, given by Equation (17) at corresponding Ha, and semi-log plots of the maximum of fluid velocity (**b**) at the gas–liquid interface (z=0), given by Equation (17), and induced MF (**c**) at the liquid–solid interface (z=−δ), given by Equation (18) for the different Ha.

The estimated values are presented in Table 3. As seen, temperature gradients $T_{x}^{\prime }$ and $T_{z}^{\prime }$ have the same order ($∼10^{7}$ K m^−1^), so for the simplification, $T_{x}^{\prime }$ can be taken instead of the $T_{z}^{\prime }$. The obtained dependencies of the fluid velocity on depth are plotted in Figure 7a. The profiles for the maximums of fluid velocity and induced MF from Ha are shown in Figure 7b,c, respectively.

As seen in Figure 7b,c, a significant fluid velocity damping and maximum possible induced MF are reached at Ha≈1.9. However, the estimated Ha for the molten pool is ∼0.2, which affects the Marangoni convection weakly (Figure 7a). Moreover, to reduce the maximum fluid velocity by at least two times, the external MF must be ∼2.6 T.

### 4.2. Microstructural and Phase Analysis

**Figure 8:**
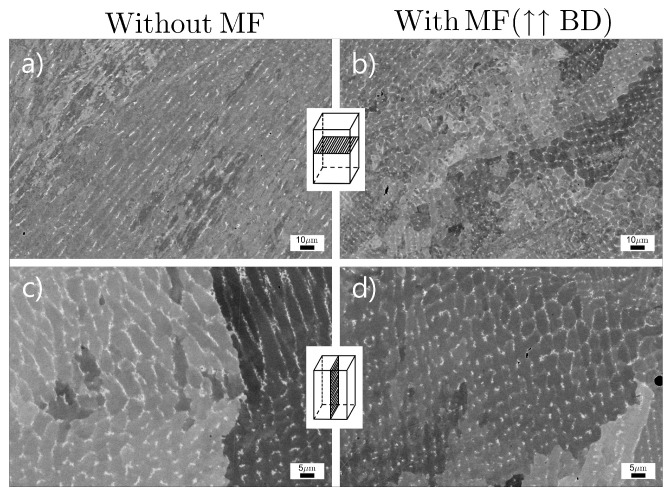
BSE images of the as-deposited Inconel 718 samples in various investigated planes: (**a**,**b**) top and (**c**,**d**) front, and no (**a**,**c**) and with (**b**,**d**) applied vertical MF. The estimated cell sizes are as follows: (**a**) 5.0 ± 1.0 μm, (**b**) 7.1 ± 1.4 μ, (**c**) 5.2 ± 1.5 μm, and (**d**) 5.2 ± 1.0 μm.

Figure 8 presents the result of the backscattered electron (BSE) image analysis for printed Inconel 718 samples under no and superimposed MF conditions. The microstructure consists of the differently shaped grains, which are formed by columnar cells. The MF influence on the cell size seems negligible and the results obtained are within the error range. For the front view, average cell size was unchanged at 5.2 μm, while for the top view, the average cell size was increased from 5.0 μm to 7.1 μm.

Du et al. [14] showed that the estimated value of the Lorentz force $F_{L}$ is $∼10^{5}$ N m^−3^, which is sufficient for the destruction of the columnar dendrites and the cause of CET. Based on current calculations, the obtained value of $F_{L}$ is higher on one order ($∼10^{6}$ N m^−3^), which should also underline the possibility of CET appearance.

According to the criterion proposed by Lehmann et al. [16], the PDAS $\delta_{1}$ depends on the TEMHD convection *U*:

(35) $$\delta_{1}=\delta_{0}{\left(1+\frac{U}{R}\right)}^{-\frac{1}{2}}$$

where $\delta_{0}$ is the primary spacing without convection (pure diffusive solute transport regime), *R* is the solidification rate. Let ξ=U/R, and then for the large ξ, the dendrite arm spacing $\delta_{1}$ should decrease significantly. Otherwise, no distinct refinement should be expected, thus $\delta_{1}=\delta_{0}$. Moreover, as mentioned in [16,20], when the diffuse-convective transition occurs (the balance of buoyancy and Lorentz force), the primary spacing increases until maximum, which identifies the planar solidification front [18]. As seen in Table 3, the Lorentz–buoyancy ratio κ is $∼10^{2}$, so the developed Lorentz force is dominant, and no diffuse- convective transition should be observed.

Nevertheless, the value of ξ can be estimated to verify the applicability of Equation (35) for AM technologies. The solidification rate *R* is proportional to the laser scan speed *V*, and the former is very high [21]. Then ξ is close to 0, and no effect of the dendrite arm spacing decrease should be seen. However, based on the experiments, the results are the opposite of what was predicted. Du et al. [14] showed that the cellular dendrite spacing was decreased in the range of error even at a small ξ$∼10^{-6}$ for the LPBF processed AlSi10Mg alloy. Simultaneously, *V* is two orders smaller for the DED technique, so assuming that *U* is the same, the PDAS should decrease more obviously. However, according to the result obtained in [5], the spacing increased when the external MF increased. The authors suggested that convection in the molten pool is the same as in the interdendritic region, which contradicts [16]. According to the qualitative results obtained in [41], the imposition of the MF causes an increase in *U* as $∼{\mathrm{MF}}^{1/2}$ at a weak MF. Therefore, this should immediately cause the dendrite arm spacing to decrease. Based on our calculations (Table 3), the order of ξ is $∼10^{-4}$, so there is no distinctive difference in the cell spacing (Figure 8c,d).

**Figure 9:**
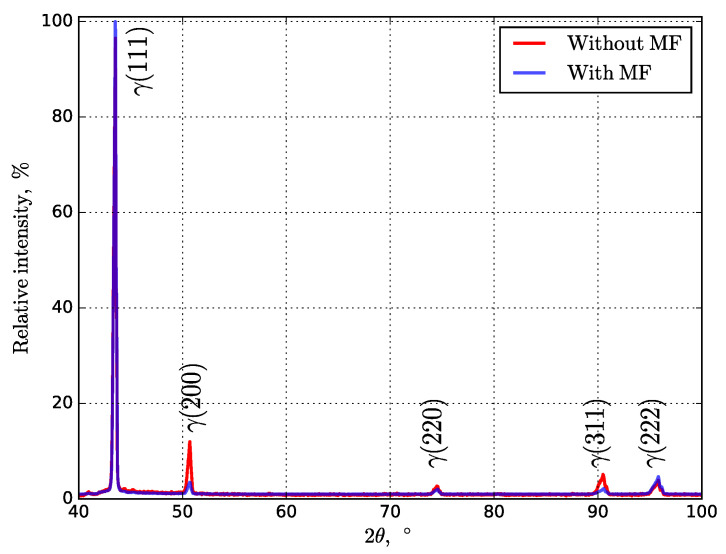
Normalized XRD patterns up to the maximum for investigated Inconel 718 samples printed under no and superimposed MF conditions.

Gamma phase is observed as the main phase in Figure 9, which is also mentioned in [4,22,42,43]. The XRD analysis revealed no presence of Ni_3_(Al, Ti), Ni_3_Nb, and intermetallic Laves ((Ni, Cr, Fe)_2_(Nb, Mo, Ti)) phases [4,44,45]. As shown in Figure 9, the intensity of the maximums varies, which can be explained by differences in texture. The diffraction line (111) presents a slightly higher relative intensity for samples printed in the MF.

**Figure 10:**
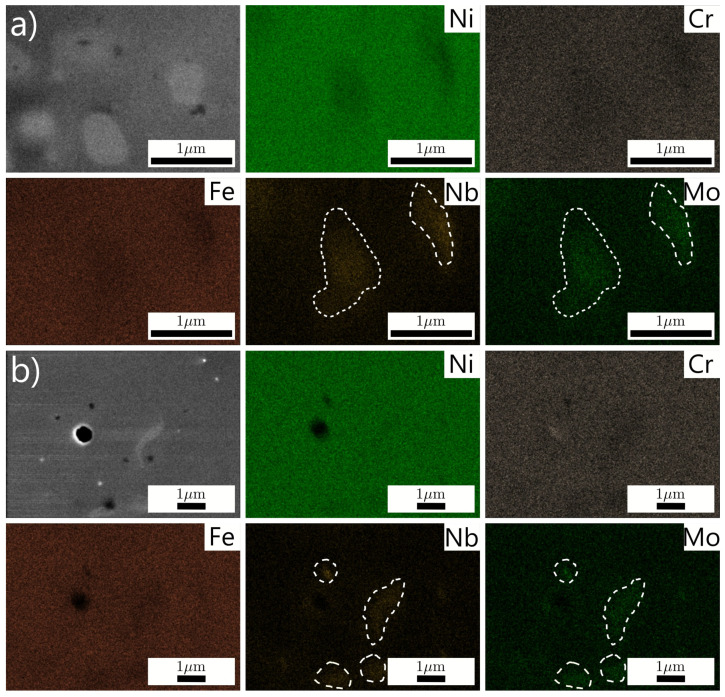
Elemental distribution maps of AM Inconel 718 samples under no (**a**) and superimposed (**b**) vertical MF conditions.

The chemical composition of the printed Inconel 718 samples under no and superimposed MF was analyzed using mapping (Table 4). It can be seen that the corresponding differences in the content of nitrogen and oxygen differ slightly, while the content of hydrogen increases twice. Although the XRD analysis result (Figure 9) did not reveal a delta phase, possibly due to its low content up to 5%, the Ni and Nb content decreased drastically, while the Fe content rose slightly when the MF was applied. According to the EDX results, almost all chemical elements seem homogeneously distributed, unlike the Nb and Mo located only in specific places (Figure 10, bright inclusion surrounded by the dashed lines) that possibly correspond to the delta phase. The accumulation of the Nb and Mo defines the growth kinetics of the formed phase, whose variable contrast is derived from the entire or partial dissolution of solid refractory metals. Therefore, microsegregation of the Nb and Mo occurs because not all elements are dissolved during solidification. The induced TEMHD convection in the interdendritic region can decrease the concentration of elements, pushing them towards the solidification front where the local temperature is higher [46]. Moreover, the estimated Pe is more than unity, leading to the prevailing convection over diffusion. Then, the probability of forming an intermetallic phase, where the Nb is the main trigger, decreases. In contrast, according to the spot analysis carried out exclusively at the cell boundaries, the average concentration of Nb and Mo was increased in an MF from 7.6 wt.% to 17.5 wt.% and from 2.9 wt.% to 3.8 wt.%, respectively. Besides, the cell boundaries were more explicit, emphasizing a more uniform ordering of Nb and Mo.

### 4.3. Porosity, Microhardness, and Mechanical Testing

**Figure 11:**
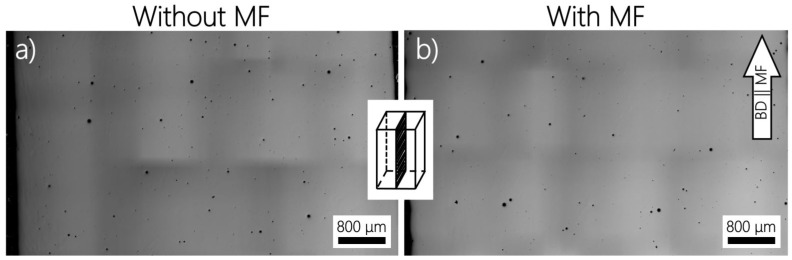
The porosity of AM Inconel 718 samples under no (**a**) and superimposed (**b**) vertical MF. The estimated pore sizes are as follows: (**a**) 36 ± 16 μm and (**b**) 41 ± 24 μm.

The experimental results of planar porosity, Vickers microhardness, and tensile tests for specimens printed in different configurations without and with MF are summarized in Table 5. The planar porosity results in the front view are illustrated in the optical micrographs (Figure 11). According to the analysis, the average porosity in the front view is decreased negligibly from 0.3% to 0.2% when exposed to an MF. However, the average pore size was slightly increased from 36 μm to 41 μm under superimposed MF. The porosity in the top view is not changed and equals 0.1%. Although the mechanisms of the pore formation are not definitively established, the observed spherical pores are dominant over irregularly shaped pores. This can identify the mechanism of porosity through the trapped gas by the initial powder feedstock and molten pool instead of the lack of fusion or keyhole mode [47,48]. Based on the predicted analytical results, the difference in magnitude of fluid velocity under the MF influence is negligible due to the smallness of Ha. Nevertheless, this is somewhat consistent with the optical micrographs (Figure 11), indicating a direct effect of fluid velocity on the final planar porosity.

The average Vickers microhardness in the front view was slightly increased from 273 HV to 278 HV under the MF influence, while in the top view was decreased from 281 HV to 267 HV. In addition, for the front view of both as-deposited samples, a specific correlation between the results of the planar porosity and Vickers microhardness was observed under no and superimposed MF conditions. The porosity was increased from the bottom to the top, while the Vickers microhardness was decreased.

**Figure 12:**
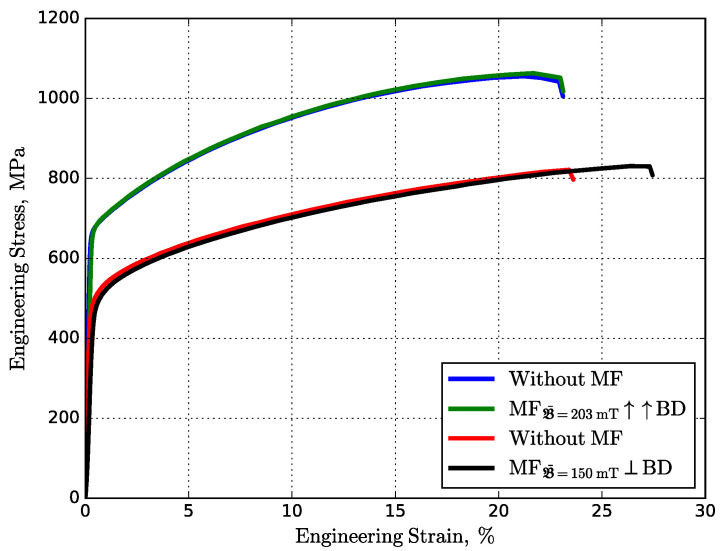
Typical engineering stress–strain curves of fabricated Inconel 718 samples obtained at different mutual orientations between the MF and build direction. (The blue/green and red/black lines correspond to the printed horizontal and vertical samples.)

The typical engineering stress–strain curves are plotted in Figure 12. The average ultimate tensile strength ($\sigma_{\mathrm{UTS}}$) of Inconel 718 samples was changed negligibly under the vertical and horizontal MF influence, from 1056 MPa to 1063 MPa and 821 MPa to 831 MPa, respectively. The apparent difference in average yield stresses ($\sigma_{Y}$) for samples fabricated vertically and horizontally, regardless of the MF, can be explained by the applied tensile load across and along the tracks. Finally, the observed average ductility (ϵ) was enhanced from 23% to 27% under the horizontal MF, which confirms a decrease in the Nb-rich precipitates in the intragranular region and an increase in the intergranular [49]. In contrast, in the case of the vertical MF, there is no difference in ductility, which can be explained by the zero Lorentz force in the mushy zone.

## 5. Conclusions

This work provides experimental results and theoretical validation for the fabricated Inconel 718 superalloy by directed energy deposition under a 0.2 T static magnetic field. According to the theoretical analysis, the induced Lorentz force dampens the Marangoni convection in the molten pool negligibly due to the small Ha, thereby slightly reducing the average planar porosity from 0.3% to 0.2%. The average Vickers microhardness was increased insignificantly for the front view under the MF and decreased by 5% for the top view. Notably, the Nb-based precipitates are reduced from 14.1 wt.% to 9.1 wt.% which can be explained by the dominant thermoelectric magnetohydrodynamic convection in the mushy zone. Finally, an average ultimate elongation was increased slightly by 4%. Although the obtained findings broaden the physical understanding of a magnetic field effect on metal 3D printing, a higher magnetic field is required to remarkably improve material performance. In addition, the solidification simulation under the TEMHD convection can provide insight into the microsegregation evolution in the interdendritic region that is crucial by means of mechanical properties.

## Figures and Tables

**Table 1:** The printing parameters recommended by InssTeK.

Parameters	Value	Unit
Powder feed rate	4.0	g min^−1^
Default laser power	375	W
Scan speed	800	mm min^−1^
Layer thickness	250	μm
Hatch spacing	500	μm
Laser spot diameter	800	μm
Shielding gas (argon)	0.01	m^3^ min^−1^
Volume energy density	225	J mm^−3^

**Table 2:** Physical properties of Inconel 718 and other parameters used for the calculations.

Physical Property	Symbol	Value	Unit
Density of liquid	$\rho_{L}$	7.40 [27] ^a^	g cm^−3^
Density of solid	$\rho_{S}$	8.19 [27] ^b^	g cm^−1^
Electrical conductivity of liquid	$\sigma_{L}$	0.72 [34]	MS m^−1^
Electrical conductivity of solid	$\sigma_{S}$	0.85 [34,35] ^b^	MS m^−1^
Dynamic viscosity	η	7.2 [27] ^a^	mPa s
Temperature coefficient of the surface tension	$\gamma^{\prime }$	−0.37 [36]	mJ m^−2^ K^−1^
Melting (liquidus) temperature	$T_{m}$	1609 [27]	K
Boiling temperature	$T_{b}$	3000 [37]	K
Solidus temperature	$T_{s}$	1533 [27]	K
Eutectic temperature	$T_{e}$	1471 [38]	K
Absolute Seebeck coefficient of the liquid	$S_{L}$	−7.08 [34] ^a^	μV K^−1^
Absolute Seebeck coefficient of the solid	$S_{S}$	−1.41 [34] ^b^	μV K^−1^
Eutectic concentration	$C_{e}$	19.1 [38]	wt.%
Initial concentration	$C_{o}$	4.7	wt.%
Diffusion coefficient	*D*	3 [39]	nm^2^ s^−1^
Vertical static MF	$B_{1}$	203	mT
Horizontal static MF	$B_{2}$	150	mT
Depth of the molten pool	δ	75 ^c^	μm
PDAS	$\delta_{1}$	10 ^d^	μm

^a^ The value is taken at the melting temperature. ^b^ The value is taken at room temperature. ^c^ Assuming that the substrate melts to ∼30% of the layer thickness at optimal laser settings [40]. ^d^ The following characteristic length scale is taken as the typical thickness of the mushy layer.

**Table 3:** The estimated quantities.

Physical Property	Symbol	Value	Unit
Temperature gradient	$T_{x}^{\prime }$	1.9 × 10^7^	K m^−1^
	$T_{z}^{\prime }$	1.3 × 10^7^	
Maximum fluid velocity	$|v_{x,\max}|$	70.9 ^a^	m s^−1^
	*U*	21 × 10^−3^ ^b^	
Maximum induced MF	$|B_{x,\max}|$	0.5 × 10^−3^ ^a^	T
Maximum induced/external MF ratio	$|B_{x,\max}|/B_{1}$	2 × 10^−3^	−
Lorentz–buoyancy ratio	κ	5 × 10^2^ ^b^	−
Hartmann number	Ha	0.2 ^a^	−
Péclet number	Pe	70 ^b^	−

^a^$\sigma_{L}$, δ, and $B_{1}$ are taken. ^b^ $\sigma_{\mathrm{eff}}$, $\delta_{1}$, $\varepsilon_{S}$ = 0.5, and $B_{2}$ are taken.

**Table 4:** Energy-dispersive X-ray elemental analysis (EDX) and nitrogen, oxygen, and hydrogen content for AM Inconel 718 samples under no and superimposed MF.

Element content, wt.%								
	Ti	Cr	Mn	Fe	Ni	Ta	Nb	Mo
No	3.8 ± 0.2	8.7 ± 0.4	1.0 ± 0.1	9.0 ± 0.5	57.4 ± 3.1	0.9 ± 0.1	14.1 ± 1.1	5.2 ± 0.6
With	3.7 ± 0.2	10.2 ± 0.5	1.0 ± 0.1	11.8 ± 0.6	51.7 ± 2.8	1.2 ± 0.1	9.1 ± 0.7	5.4 ± 0.6
Element content, 10^−4^ wt.%								
				N_2_	O_2_	H_2_		
			No	42	45	0.2		
			With	40	45	0.5		

**Table 5:** Measured planar porosity, Vickers microhardness, and tensile strength values for fabricated Inconel 718 horizontal and vertical specimens under no and superimposed MF conditions.

	Porosity, %		Hardness, HV		$\sigma_{\mathrm{UTS}}$, MPa		$\sigma_{Y}$, MPa		ϵ, %	
	Top	Front	Top	Front	Hor	Vert	Hor	Vert	Hor	Vert
No	0.1	0.3 ± 0.1	281 ± 11	273 ± 14	1056 ± 11	821 ± 22	662 ± 5	511 ± 47	23 ± 1	23 ± 5
With	0.1	0.2 ± 0.1	267 ± 14	278 ± 19	1063 ± 2	831 ± 31	669 ± 14	499 ± 25	23 ± 1	27 ± 7

## Data Availability

No new data were created or analyzed in this study. Data sharing is not applicable to this article.

## References

[1] Gibson I., Rosen D., Stucker B. (2015). Additive Manufacturing Technologies: 3D Printing, Rapid Prototyping, and Direct Digital Manufacturing.

[2] Piscopo G., Atzeni E., Salmi A. (2019). A hybrid modeling of the physics-driven evolution of material addition and track generation in laser powder directed energy deposition. Materials.

[3] Piscopo G., Salmi A., Atzeni E. (2021). Influence of High-Productivity Process Parameters on the Surface Quality and Residual Stress State of AISI 316L Components Produced by Directed Energy Deposition. J. Mater. Eng. Perform..

[4] Wang J., Wang Y., Shi J., Su Y. Effect of external magnetic field on the microstructure of 316L stainless steel fabricated by directed energy deposition. Proceedings of the ASME 2019 International Mechanical Engineering Congress and Exposition.

[5] Du D., Dong A., Shu D., Wang D., Zhu G., Sun B., Lavernia E.J. (2020). Influence of Static Magnetic Field on the Microstructure of Nickel-Based Superalloy by Laser-Directed Energy Deposition. Metall. Mater. Trans. A Phys. Metall. Mater. Sci..

[6] Kao A., Gan T., Tonry C., Krastins I., Pericleous K. (2020). Study of thermoelectric magnetohydrodynamic convection on solute redistribution during laser additive manufacturing. IOP Conf. Ser. Mater. Sci. Eng..

[7] Todaro C.J., Easton M.A., Qiu D., Zhang D., Bermingham M.J., Lui E.W., Brandt M., StJohn D.H., Qian M. (2020). Grain structure control during metal 3D printing by high-intensity ultrasound. Nat. Commun..

[8] Chen Q., Zhao Y., Strayer S., Zhao Y., Aoyagi K., Koizumi Y., Chiba A., Xiong W., To A.C. (2021). Elucidating the effect of preheating temperature on melt pool morphology variation in Inconel 718 laser powder bed fusion via simulation and experiment. Addit. Manuf..

[9] Saldi Z.S. (2012). Marangoni Driven Free Surface Flows in Liquid Weld Pools. Ph.D. Thesis.

[10] Leung C.L.A., Marussi S., Atwood R.C., Towrie M., Withers P.J., Lee P.D. (2018). In situ X-ray imaging of defect and molten pool dynamics in laser additive manufacturing. Nat. Commun..

[11] Xia M., Gu D., Yu G., Dai D., Chen H., Shi Q. (2016). Selective laser melting 3D printing of Ni-based superalloy: Understanding thermodynamic mechanisms. Sci. Bull..

[12] Zhao Y., Aoyagi K., Yamanaka K., Chiba A. (2020). Role of operating and environmental conditions in determining molten pool dynamics during electron beam melting and selective laser melting. Addit. Manuf..

[13] Tohru M., Ichiro T. (1988). Effect of magnetic field on onset of Marangoni convection. Int. J. Heat Mass Transf..

[14] Du D., Haley J.C., Dong A., Fautrelle Y., Shu D., Zhu G., Li X., Sun B., Lavernia E.J. (2019). Influence of static magnetic field on microstructure and mechanical behavior of selective laser melted AlSi10Mg alloy. Mater. Des..

[15] Shercliff J.A. (1979). Thermoelectric magnetohydrodynamics. J. Fluid Mech..

[16] Lehmann P., Moreau R., Camel D., Bolcato R. (1998). A simple analysis of the effect of convection on the structure of the mushy zone in the case of horizontal Bridgman solidification comparison with experimental results. J. Cryst. Growth.

[17] Lehmann P., Moreau R., Camel D., Bolcato R. (1998). Modification of interdendritic convection in directional solidification by a uniform magnetic field. Acta Mater..

[18] Kurz W., Fisher D.J. (1986). Fundamentals of Solidification.

[19] Strickland J., Nenchev B., Dong H. (2020). On directional dendritic growth and primary spacing—A review. Crystals.

[20] Dupouy M.D., Camel D., Favier J.J. (1992). Natural convective effects in directional dendritic solidification of binary metallic alloys: Dendritic array primary spacing. Acta Metall. Mater..

[21] Wang Y., Shi J. (2019). Texture control of Inconel 718 superalloy in laser additive manufacturing by an external magnetic field. J. Mater. Sci..

[22] Liu F., Cheng H., Yu X., Yang G., Huang C., Lin X., Chen J. (2018). Control of microstructure and mechanical properties of laser solid formed Inconel 718 superalloy by electromagnetic stirring. Opt. Laser Technol..

[23] Landau L.D., Pitaevskii L.P., Lifshitz E.M. (1984). Electrodynamics of Continuous Media: Course of Theoretical Physics. Volume 8.

[24] Landau L.D., Lifshitz E.M. (1987). Fluid Mechanics: Course of Theoretical Physics. Volume 6.

[25] Shercliff J.A. (1966). A Textbook of Magnetohydrodynamics.

[26] Alboussiere T., Moreau R., Camel D. (1991). Influence of a Magnetic Field on the Solidification of Metallic Alloys. Comptes Rendus I’Academie des Science.

[27] Valencia J.J., Quested P.N. (2008). Thermophysical Properties, ASM Handbook. ASM.

[28] Poirier D.R. (1987). Permeability for flow of interdendritic liquid in columnar-dendritic alloys. Metall. Trans. B.

[29] Chamsri K., Bennethum L.S. (2015). Permeability of fluid flow through a periodic array of cylinders. Appl. Math. Model..

[30] Callister W. (1991). Materials science and engineering: An introduction (2nd edition). Mater. Des..

[31] Zhang C., Jahazi M., Gallego P.I. (2020). On the impact of microsegregation model on the thermophysical and solidification behaviors of a large size steel ingot. Metals.

[32] Chang S., Stefanescu D.M. (1996). A model for macrosegregation and its application to Al-Cu castings. Metall. Mater. Trans. Phys. Metall. Mater. Sci..

[33] Beckermann C., Gu J.P., Boettinger W.J. (2000). Development of a freckle predictor via Rayleigh number method for single-crystal nickel-base superalloy castings. Metall. Mater. Trans. A Phys. Metall. Mater. Sci..

[34] McElroy D.L., Williams R.K., Moore J.P., Graves R.S., Weaver F.J. (1978). The Physical Properties of Inconel Alloy 718 from 300 to 1000 K BT-Thermal Conductivity 15.

[35] Butts D.A., Gale W.F. (2003). Equilibrium diagrams. Smithells Metals Reference Book.

[36] Knapp G.L., Raghavan N., Plotkowski A., DebRoy T. (2019). Experiments and simulations on solidification microstructure for Inconel 718 in powder bed fusion electron beam additive manufacturing. Addit. Manuf..

[37] Cao L., Yuan X. (2019). Study on the Numerical Simulation of the SLM Molten Pool Dynamic Behavior of a Nickel-Based Superalloy on the Workpiece Scale. Materials.

[38] Knorovsky G.A., Cieslak M.J., Headley T.J., Romig A.D., Hammetter W.F. (1989). INCONEL 718: A solidification diagram. Metall. Trans. A.

[39] Nastac L., Stefanescu D.M. (1996). Macrotransport-solidification kinetics modeling of equiaxed dendritic growth: Part II. Computation problems and validation on INCONEL 718 superalloy castings. Metall. Mater. Trans. A.

[40] Chakraborty S.S., Dutta S. (2019). Estimation of dilution in laser cladding based on energy balance approach using regression analysis. Sadhana Acad. Proc. Eng. Sci..

[41] Li X., Fautrelle Y., Ren Z. (2007). Influence of thermoelectric effects on the solid-liquid interface shape and cellular morphology in the mushy zone during the directional solidification of Al-Cu alloys under a magnetic field. Acta Mater..

[42] Mukhtarova K.S., Shakhov R.V., Mukhtarov S.K., Smirnov V.V., Imayev V.M. (2019). Microstructure and mechanical properties of the inconel 718 superalloy manufactured by selective laser melting. Lett. Mater..

[43] Shakhov R.V., Mukhtarova K.S. (2018). Nb rich precipitates in Inconel 718 produced by selective laser melting. Lett. Mater..

[44] Caliari F.R., Guimarães N.M., Reis D.A.P., Couto A.A., De Moura Neto C., Candioto K.C.G. (2013). Study of the secondary phases in Inconel 718 aged superalloy using thermodynamics modeling. Key Eng. Mater..

[45] Yong C.K., Gibbons G.J., Wong C.C., West G. (2020). A Critical Review of the Material Characteristics of Additive Manufactured IN718 for High-Temperature Application. Metals.

[46] Chen Y., Guo Y., Xu M., Ma C., Zhang Q., Wang L., Yao J., Li Z. (2019). Study on the element segregation and Laves phase formation in the laser metal deposited IN718 superalloy by flat top laser and gaussian distribution laser. Mater. Sci. Eng. A.

[47] Ng G.K.L., Jarfors A.E., Bi G., Zheng H.Y. (2009). Porosity formation and gas bubble retention in laser metal deposition. Appl. Phys. A Mater. Sci. Process..

[48] Wolff S.J., Wang H., Gould B., Parab N., Wu Z., Zhao C., Greco A., Sun T. (2021). In situ X-ray imaging of pore formation mechanisms and dynamics in laser powder-blown directed energy deposition additive manufacturing. Int. J. Mach. Tools Manuf..

[49] Anderson M., Thielin A.L., Bridier F., Bocher P., Savoie J. (2017). *δ* Phase precipitation in Inconel 718 and associated mechanical properties. Mater. Sci. Eng. A.

